# Reporting of Postprotocol Therapies and Attrition in Multiple Myeloma Randomized Clinical Trials: A Systematic Review

**DOI:** 10.1001/jamanetworkopen.2021.8084

**Published:** 2021-04-28

**Authors:** Ghulam Rehman Mohyuddin, Kelly Koehn, Al-Ola Abdallah, Aaron M. Goodman, Vinay Prasad

**Affiliations:** 1 Department of Hematological Malignancies and Cellular Therapeutics, Kansas University Medical Center, Kansas City; 2 Division of Blood and Marrow Transplantation, University of California, San Diego, La Jolla; 3 Divisions of Hematology & Medical Oncology, University of California, San Francisco

## Abstract

**Importance:**

A thorough understanding of the optimal role and sequence of agents for treatment of multiple myeloma (MM) requires knowledge of the use and rate of postprotocol therapies in randomized clinical trials (RCTs).

**Objectives:**

To examine the proportion of MM RCTs that reported postprotocol therapies and, among those, the percentage of patients who received no further therapy and how treatments differed between the control and intervention arms.

**Evidence Review:**

The reporting of postprotocol therapies was systematically assessed in published MM RCTs using 3 databases (PubMed, Embase, and Cochrane Registry of Controlled Trials) for MM RCTs from January 1, 2005, to December 30, 2019. All MM RCTs were included, and all other studies, such as editorials, nonrandomized studies, and review articles, were excluded.

**Findings:**

A total of 103 RCTs were identified (47 251 patients); of these, 45 (43.7%) reported subsequent treatments in that publication or in any subsequent publication. Trials funded by pharmaceutical companies (26 of 47 [55.3%]) were more likely to report subsequent treatments than cooperative group studies (19 of 56 [33.9%]) (χ^2^_1,103_ = 4.8; *P* = .03). Differences were found in the treatments received between the intervention and control arms of RCTs. When data were reported, 5150 of 9351 patients (54.9%) in RCTs of newly diagnosed MM and 2197 of 4501 patients (48.8%) in RCTs of relapsed/refractory MM received any subsequent therapy.

**Conclusions and Relevance:**

Postprotocol therapies in MM RCTs are often not reported and, when they are, many patients receive no further therapy. Reporting guidelines for postprotocol therapies are needed.

## Key Points

**Question:**

What proportion of multiple myeloma randomized clinical trials report postprotocol therapies, and when reported, how do these therapies compare with existing standard of care?

**Findings:**

In this systematic review of 103 randomized clinical trials including 47 251 patients, only 43.7% of the trials reported postprotocol therapies. When described, the proportion of patients receiving postprotocol therapies was low, and often not at par with standard of care therapy.

**Meaning:**

These findings suggest that reporting of postprotocol therapies is poor in multiple myeloma trials, necessitating reporting guidelines on postprotocol therapies for ongoing and future trials.

## Teaser

This systematic review examines the reporting of postprotocol treatment received by patients enrolled in randomized clinical trials for new-onset or relapsed/refractory multiple myeloma.

## Introduction

Advances in the treatment of multiple myeloma (MM) have led to improved survival.^1^ Triplet therapy is recommended at diagnosis and relapse, because multiple randomized clinical trials (RCTs) have demonstrated the superiority of triplet over doublet therapy with respect to efficacy outcomes, such as progression-free survival (PFS) and overall survival (OS).^2,3,4^ To our knowledge, there has not been a systematic review of reporting on postprotocol therapies of patients in these trials. An understanding of the rate and quality of postprotocol therapies is necessary to assess whether combined use of multiple agents is superior to the sequential use of these agents in accordance with the best available standard of care before the study.^5^ Some contend that, in the absence of improvements in OS or quality of life, gains in PFS alone do not justify the use of drugs in combination that were previously used in sequence.^6,7^

For these reasons, we sought to systematically assess the reporting of postprotocol therapies in MM RCTs published from 2005 to 2019. We also aimed to determine what fraction of patients received no further therapies in trials in which those data were reported.

## Methods

### Search Strategy

We performed a search of 3 databases: (MEDLINE/PubMed, Embase, and Cochrane Registry of Controlled Trials). An example search strategy using Embase is highlighted in the eTable in the Supplement. Two of us (G.R.M. and K.K.) independently screened all studies and any conflict was resolved through mutual discussion. This systematic review was performed according to the Preferred Reporting Items for Systematic Reviews and Meta-analyses (PRISMA) reporting guideline.^8^

Our search strategy was restricted to include RCTs that were published in manuscript or abstract form from January 1, 2005, to December 30, 2019. The search was last updated on April 1, 2020. All other publications, including editorials, case reports, case series, review articles, case-control studies, retrospective/prospective cohort studies, and single-arm studies, were excluded. Furthermore, trials not evaluating therapeutic interventions, such as those focusing solely on supportive care measures, infection mitigation preventions, or different stem cell mobilization strategies, were excluded. The search strategy was not restricted by language. Abstracts from conference proceedings that were captured on these databases via our search strategy, such as those on Embase, were also included.

Two of us (G.R.M. and K.K.) performed and verified all data extraction. Extracted data were tabulated using Microsoft Excel (Microsoft Corp). We identified the disease phase (relapsed/refractory or frontline) and location of study (enrollment in the US alone vs multinational). The most recent article or abstract with updated data was used to collect data for each study.

The primary outcomes were the proportion of RCTs that reported postprotocol therapies and, in trials that reported postprotocol therapies, the percentage of patients who received no further therapy.

For quantitative estimation of attrition and receipt of subsequent therapy, we only included studies that clearly reported the number of patients receiving subsequent therapies in both the control and intervention arm. Studies that only reported on a particular type of therapy or only on one arm were excluded from the quantitative analysis.

### Statistical Analysis

Analysis with χ^2^ testing was used to calculate differences in proportions between trials funded by pharmaceutical companies vs cooperative group studies, whenever applicable. Significance testing was 2 sided, and differences at *P* < .05 were considered significant. Data analysis was performed using SPSS, version 26 (IBM).

## Results

After excluding duplicate RCTs and trials that did not meet the inclusion criteria, a total of 103 discrete RCTs were identified (47 251 patients) (Figure). Table 1 highlights the characteristics of the included studies.

**Figure.**
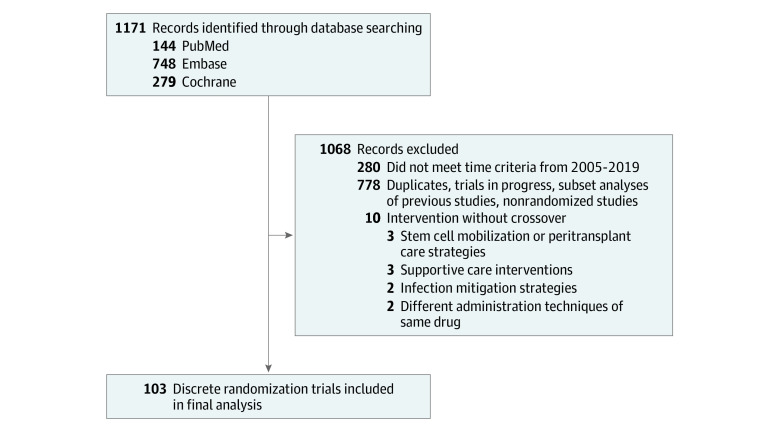
Flow Diagram

**Table 1.** Characteristics of 103 Included Studies

Study Characteristic	No. of studies (%)
Pharmaceutical company funded	47 (45.6)
Cooperative group/single-center (not pharmaceutical company)	56 (54.4)
Frontline or consolidation after frontline	64 (62.1)
Relapsed/refractory	33 (32.0)
Maintenance	6 (5.8)
Multinational	60 (58.3)
Limited to the US	16 (15.5)

We found 45 of the 103 RCTs (43.7%) that reported subsequent treatments in the original article or in any follow-up publication or abstract. Among these 45 trials, the subsequent treatments were reported in the main article in 11 studies (24.4%), in a supplemental appendix in 11 studies (24.4%), and in a subsequent abstract presentation in 23 studies (51.1%).

Among 47 pharmaceutical company–funded RCTs, postprotocol therapies were reported in 26 (55.3%). Among 56 cooperative group RCTs, postprotocol therapies were reported in 19 (33.9%). This difference was statistically significant (χ^2^_1,103_ = 4.8; *P* = .03).

### Attrition Rates

Among the 45 RCTs that reported subsequent therapies, 27 (60.0%) reported clearly on the number of patients in both the control and intervention arms who subsequently received therapy. Among 7665 patients in the intervention arms of these trials, only 3845 (50.2%) received a subsequent (nonmaintenance) line of therapy. Among 6801 patients in the control arms of these trials, 3793 (55.8%) eventually received a subsequent line of therapy.

We stratified receipt of subsequent therapy for frontline and relapsed/refractory trials. In RCTs of newly diagnosed MM, subsequent systemic treatment was received by 2472 of 4248 (58.2%) patients enrolled in the control arms and 2668 of 5103 (52.3%) patients in the intervention arms (overall, 5150 of 9351 [54.9%]). In RCTs of relapsed/refractory MM, subsequent systemic treatment was received by 1148 of 2246 (51.1%) patients enrolled in the control arms and 1049 of 2255 (46.5%) patients in the intervention arms (overall, 2197 of 4501 [48.8%]). Consequently, 45.1% of patients in newly diagnosed MM RCTs and 51.2% of patients in the relapsed/refractory RCT setting received no further therapy.

Among the 27 RCTs that reported clearly on the number of patients receiving subsequent therapies, 13 were funded by pharmaceutical companies (48.1%) and 14 were cooperative group studies (51.9%). Among the 13 pharmaceutical company–funded studies, 4187 of 8375 patients (50.0%) received subsequent therapies, whereas in the 14 cooperative group studies, 3451 of 6091 patients (56.7%) received subsequent therapies (*P* < .001).

### Translation of a PFS to an OS Benefit

In 45 RCTs (43.7%) in which subsequent treatment was clearly reported, a PFS benefit was reported in 38 (84.4%) of these trials. Based on the most recent follow-up of patients, this PFS benefit translated to an OS benefit in 17 of RCTs (44.7% of those that reported a PFS advantage) and had not yet resulted or did not result in a benefit in the remaining trials (21 [55.3%]). In 59 RCTs that did not report subsequent treatment, 22 (37.3%) described a PFS and an OS benefit was seen in 5 trials (22.7% of trials that reported a PFS benefit).

### Postprotocol Treatments for Pivotal Trials

We assessed the description of subsequent treatments for major practice-changing trials over the past 15 years. Table 2 highlights pivotal trials in the frontline setting with respect to their reporting and practices of postprotocol treatments.^4,9,10,11,12,13,14^ Receipt of daratumumab post progression was low in the MAIA^9^ and ALCYONE^10^ trials. The SWOG 0777 trial, which evaluated combined bortezomib, lenalidomide, and dexamethasone vs combined lenalidomide and dexamethasone, did not report on subsequent treatments in a follow-up publication; hence, it is unknown what proportion of patients randomized to lenalidomide and dexamethasone received bortezomib at progression of MM.^4^

**Table 2.** Pivotal Frontline Trials and Their Reporting/Description of Postprotocol Treatments

Trial	Enrolled in US	Intervention/control	Subsequent treatment reported	OS advantage reported	Magnitude of OS advantage reported		Observations regarding subsequent treatment
					Median	HR (95% CI)	
SWOG 0777^4^	Yes	Bortezomib, lenalidomide, dexamethasone vs lenalidomide, dexamethasone	No	Yes	NR vs 69 mo	0.71 (0.54-0.93)	NA
MAIA^9^	Yes	Dexamethasone, lenalidomide, daratumumab vs lenalidomide, dexamethasone	Yes	No	NA	NA	None of the 3 most common regimens used at progression (bortezomib; bortezomib, cyclophosphamide, dexamethasone; bortezomib, melphalan, prednisone) for control arm were daratumumab-containing regimens
ALCYONE^10^	No	Daratumumab, bortezomib, melphalan, prednisone vs bortezomib	Yes	Yes	36-mo OS, 78% vs 67.9%	0.60 (0.46-0.80)	Only 10% of patients in control arm received a daratumumab-containing regimen at first progression
VISTA^11^	Yes	Bortezomib, melphalan, prednisone vs melphalan, prednisone	Yes	Yes	56.4 vs 43.1 mo	0.70 (Not reported) *P* = .0004	43% Of patients in melphalan, prednisone arm received subsequent bortezomib
MM-015^12^	No	Melphalan, prednisone, lenalidomide vs melphalan, prednisone vs melphalan, prednisone, lenalidomide continuously	Yes	No OS	NA	NA	61.7% Of patients in melphalan, prednisone, arm received subsequent lenalidomide
Rajkumar et al^13^	Yes	Lenalidomide, low-dose dexamethasone vs high-dose dexamethasone	Yes	Yes (at 1- and 2-y mark)	1-y OS: 96% (range, 94%-99%) vs 87% (range, 82%-92%)	NA	All patients crossed over to low-dose dexamethasone when clear signal of survival benefit was seen, and the survival curves merged at 3-y mark
FIRST^14^	Yes	Lenalidomide, dexamethasone vs melphalan, prednisone, thalidomide	Yes	Yes	59.1 vs 49.1 mo	0.78 (0.67-0.92)	Similar treatments at progression, bortezomib-based regimen most commonly used

Abbreviations: HR, hazard ratio; NA, not applicable; NR, not reached; OS, overall survival.

Table 3 highlights the reporting and practices of postprotocol treatments in pivotal trials evaluating the relapsed/refractory MM setting.^2,3,15,16,17,18,19^ In pivotal triplet vs doublet therapy trials, such as ASPIRE (carfilzomib, lenalidomide, and dexamethasone)^2^ and ELOQUENT-2 (elotuzumab, lenalidomide, and dexamethasone vs lenalidomide and dexamethasone),^3^ use of carfilzomib and elotuzumab was low in the control arm. Conversely, most patients in the control arm of the CASTOR trial (daratumumab/bortezomib/dexamethasone vs bortezomib/dexamethasone) received daratumumab at progression.^15^

**Table 3.** Pivotal Relapsed/Refractory Trials and Their Reporting and Description of Postprotocol Treatments

Trial	Enrolled in US	Intervention/control	Subsequent treatment reported	OS advantage reported	Magnitude of OS advantage reported		Observations regarding subsequent treatment
					Median	HR (95% CI)	
CASTOR^15^	Yes	Daratumumab, bortezomib, dexamethasone vs bortezomib, dexamethasone	Yes	Not at this time	NA		Subsequent treatment not reported in updated publications other than 81 patients in bortezomib arm, dexamethasone arm received daratumumab monotherapy at progression^16,17^
POLLUX^18^	Yes	Daratumumab, lenalidomide, dexamethasone vs lenalidomide, dexamethasone	Yes	Not yet	NA		Most patients with MM progression (77.8%) in lenalidomide, dexamethasone arm received daratumumab monotherapy at progression
ASPIRE^2^	Yes	Carfilzomib, lenalidomide, dexamethasone vs lenalidomide, dexamethasone	Yes	Yes	48.3 vs 40.4 mo	0.79 (0.67-0.95)	Only 2% of patients in lenalidomide, dexamethasone arm received carfilzomib subsequently
ELOQUENT-2^3^	Yes	Elotuzumab, lenalidomide, dexamethasone vs lenalidomide, dexamethasone	Yes	Yes	48.3 vs 39.6 mo	0.82 (0.68-1.00)	Elotuzumab not given to control arm on progression
ENDEAVOR^19^	Yes	Carfilzomib, dexamethasone vs bortezomib, dexamethasone	Yes	Yes	47.6 vs 40 mo	0.79 (0.65-0.96)	Only 8% of patients in bortezomib arm received carfilzomib subsequently

Abbreviations: MM, multiple myeloma; NA, not applicable; OS, overall survival.

## Discussion

Despite treatment advances, most patients with MM relapse. Hence, combinations of treatments are attempted in sequence to reduce tumor burden, improving quality and quantity of life. The clinical question of whether to use the available armamentarium of drugs early in the course or to reserve them for sequential administration is relevant. To understand the value of a combination in improving overall survival, an overall accurate reporting of crossover and postprotocol therapies is required.

Our systematic review noted that reporting of postprotocol therapies for MM RCTs is low, occurring only in 43.7% of trials. Because subsequent therapies were not reported line by line, we were only able to determine whether patients received any subsequent therapy—not how many lines of therapies they subsequently received. Even for pivotal practice-changing trials, such as SWOG 0777, which established the efficacy of combined bortezomib, lenalidomide, and dexamethasone as frontline treatment for MM, postprotocol treatments were not reported in the follow-up publication.^4^ The reporting rate for postprotocol therapies was significantly lower in cooperative group studies (33.9%) compared with pharmaceutical company–funded RCTs (55.3%). This difference is likely related to budgetary constraints of cooperative group trials^20^ and deserves further study across different diseases. Conversely though, when reported, receipt of postprotocol therapies was higher in cooperative group studies than in pharmaceutical studies, a finding that may reflect the settings in which the trial enrolled patients.

It is undeniable that the new agents available for MM have led to markedly improved survival, as reported both by contemporary clinical trial data and real-world observational data.^21,22,23^ It is also true that this improvement in survival is associated both with increased use of combination therapies and availability of newer agents. However the current literature leaves considerable uncertainty regarding the optimal sequence, despite trials showing survival gains from triplet over doublet therapy, because rates of postprotocol therapies cannot be assessed.

In most of the trials we studied, a PFS advantage was not translated to an OS advantage, which is likely due to the abundance of options available for treatment after progression. Upon longer follow-up, an overall survival benefit may yet be established. Poor reporting of postprotocol therapies raises the question of whether use of combination therapy rather than sequencing of multiple agents truly improves survival or overall quality of life.

Crossover is desirable when determining whether a drug used in a latter line of therapy should be moved up front.^5^ As our review suggests, the low rates of subsequent use of elotuzumab and carfilzomib in the ELOQUENT-2 (elotuzumab, lenalidomide, and dexamethasone vs lenalidomide and dexamethasone)^2^ and ASPIRE (carfilzomib, lenalidomide, and dexamethasone vs lenalidomide and dexamethasone)^3^ studies in the control arm demonstrate that these agents prolong survival for patients with MM—a finding that may not have been noted had more patients received these agents in later lines of treatment in these trials. Conversely though, the poor access to these medications upon MM progression in the control arm also makes it difficult to interpret the true value of triplet over doublet therapy, because these trials can no longer answer whether a triplet strategy incorporating the novel drug in question is better than a doublet strategy in which access to these medications is provided at MM progression. We acknowledge that elotuzumab has no single agent activity and that at progression it would have to be combined with other agents. The other contemporary triplet vs doublet therapy relapsed/refractory trials, such as OPTIMISMM (pomalidomide, bortezomib, and dexamethasone vs bortezomib and dexamethasone),^24^ POLLUX (daratumumab, lenalidomide, and dexamethasone vs lenalidomide and dexamethasone),^18^ and CASTOR (daratumumab, bortezomib, and dexamethasone vs bortezomib and dexamethasone)^15^ have not yet reported an OS advantage, and follow-up continues. There was relatively high use of daratumumab at MM progression in the POLLUX trial (77.8% of patients with MM progression in the lenalidomide and dexamethasone arm received daratumumab at progression), and, owing to crossover, an OS advantage may not be seen.^18^

When evaluating the true use of moving an agent with established efficacy in later-line treatments to an earlier line of treatment, access to the treatment on MM progression should be ensured and the trial should be powered for OS.^5^ Although the ALCYONE trial evaluating the addition of daratumumab to up-front therapy of bortezomib, melphalan, and prednisone has already demonstrated an OS benefit,^10^ only 10% of patients in the control arm received a daratumumab-containing regimen at first relapse. Two important caveats must thus be noted. First, this induction regimen is not used in the US, and second, almost all patients who have not received daratumumab as initial therapy are now expected to receive daratumumab at progression in accordance with guidelines.^1^ Thus, the utility of addition to daratumumab to standard up-front therapy in terms of improving overall survival remains unknown. Data from the GRIFFIN trial evaluating daratumumab, lenalidomide, bortezomib, and dexamethasone vs lenalidomide, bortezomib, and dexamethasone are immature and the trial is not powered for OS^25^; however, ongoing trials, such as PERSEUS,^26^ must ensure that daratumumab is given on MM progression and demonstrate an OS benefit to determine the true value of adding daratumumab to first-line therapy. Another commonly used regimen for newly diagnosed MM in the US is daratumumab, lenalidomide, and dexamethasone. Although data on OS are not mature, recently updated data from the MAIA trial evaluating this regimen showed that none of the 3 most commonly used regimens after MM progression for patients in the control group contained daratumumab, and all regimens were thus below the US standard of care.^9^

When transparent reporting of postprotocol therapies is demonstrated, clinicians and patients can make informed decisions of the true value and sequencing of a treatment. One example is the IFM 2009 trial, where transparent reporting of postprotocol therapies allowed for optimal decision-making on the role and timing of an autologous stem cell transplant for patients with myeloma.^27^ Another example is the long-term follow-up of the GIMEMA-MMY-3006 study,^28^ which compared bortezomib, thalidomide, and dexamethasone vs thalidomide and dexamethasone as induction therapy, followed by double autologous stem cell transplant for newly diagnosed MM. With a median follow-up of 124.1 months and transparent reporting of balanced subsequent therapies in both arms, there was a clinically meaningful OS benefit for patients receiving triplet therapy induction vs a doublet therapy induction of thalidomide and dexamethasone, highlighting the value of triplet therapy as induction.

We noted that many patients in these RCTs received no further therapy. Although reporting was inconsistent, among the trials that reported on receipt of subsequent therapy, only 54.9% of patients in frontline trials and 48.8% of patients in relapsed/refractory trials went on to receive any further line of therapy. This finding is similar to an analysis of real-world data using a nationwide database in which Fonseca et al^29^ reported high rates of attrition. In that study, only approximately half of the patients with newly diagnosed MM not undergoing transplant at diagnosis received a subsequent line of therapy. Considering that clinical trial patient populations are healthier than real-world patient populations and are often at institutions that have ready access to treatments if relapse occurs,^30^ the receipt of postprotocol therapy in our study should exceed levels seen in routine clinical practice and raises the concern that the low level reflects the limited availability of agents in multinational trial settings. This rate of attrition has led to calls to intensify the first treatment given to patients, because it may be the last treatment they receive^29^; however, it is unknown whether intensifying treatments alone would improve outcomes in settings where access to subsequent lines of treatment is possible or whether intensifying treatment would merely add to toxic effects.

### Limitations

There are limitations to our study. Our search strategy included only RCTs published by December 2019. As a result, inferences on more recent practice-changing studies cannot be made. Conversely, studies that reported results in 2018 or 2019 may not have had adequate follow-up at the time our analysis was done and may yet go on to report postprotocol therapies in subsequent publications. The denominator we used for rates of subsequent therapy was based on enrolled patients, and not patients who experienced progressive MM but remained alive and able to tolerate therapy. Choice of this denominator was a necessity because trials did not report exactly how many patients had progressed and were still alive and not lost to follow-up to be eligible to receive more therapy. Clearly reporting the percentage of PFS events that were death (rather than progression) would help in future analyses. Most trial protocols do not specify posttrial therapy, so any subsequent treatments the patients receive are left to the discretion of the physician and not random assignment. Without the availability of patient-level data, it is difficult to assess exactly why the amount of postprotocol therapy was low. For patients who received no further treatment, the assessing physician may have determined no further treatment was of benefit. Owing to a lack of patient-level data, we cannot analyze the disease course and further treatment of patients who discontinued trial therapy because of progression of disease vs those who had favorable results with the trial therapy. Future RCTs can address this issue by using a dynamic treatment regimen strategy in which patients are continually randomized at decision points after the start of treatment. As an example, patients who experience adverse events are randomized to a certain strategy, whereas those who experience relapse are randomized to a different set of strategies.^31^

## Conclusions

Findings in this study noted poor reporting of postprotocol therapies in MM RCTs and a high percentage of patients who received no therapy when trials report these percentages. Discrepancies between the postprotocol therapies between the 2 arms or between the therapy received and the prevailing standard of care occurred in several trials and hence precludes an understanding of the true value of treatments. Transparent reporting of postprotocol therapies should be emphasized in ongoing and future studies.

## Notes

Supplement. **eTable.** Example Search Strategy
