# Supplementary material for: Reporting of Postprotocol Therapies and Attrition in Multiple Myeloma Randomized Clinical Trials: A Systematic Review
Source: JAMA Netw Open. 2021 Apr 28;4(4):e218084. doi: 10.1001/jamanetworkopen.2021.8084 (PMC8082314; doi:10.1001/jamanetworkopen.2021.8084)
Supplement: Supplement. — eTable. Example Search Strategy [file jamanetwopen-e218084-s001.pdf]

Supplementary Online Content

Mohyuddin GR, Koehn K, Abdallah AO, Goodman AM, Prasad V. Reporting of postprotocol therapies and attrition in multiple myeloma randomized clinical trials: a systematic review. *JAMA Netw Open*. 2021;4(4):e218084. doi:10.1001/jamanetworkopen.2021.8084

eTable. Example Search Strategy

This supplementary material has been provided by the authors to give readers additional information about their work.

© 2021 Mohyuddin GR et al. *JAMA Network Open.*

eTable. Example Search Strategy

S. No.	Query	Studies
1.	myeloma OR myelomas OR myelomatos* OR 'kahler disease' OR 'myeloma'/exp	108931
2.	(myeloma OR myelomas OR myelomatos* OR 'kahler disease' OR 'myeloma'/exp) AND [2005-2020]/py	74264
3.	#2 AND 'human'/de	66345
4.	#2 AND 'human'/de NOT ('conference review'/it OR 'editorial'/it OR 'review'/it OR 'short survey'/it)	55869
5.	'phase 3 clinical trial'/exp	44510
6.	'crossover procedure':de OR 'double-blind procedure':de OR 'randomized controlled trial':de OR 'single-blind procedure':de OR random*:de,ab,ti OR factorial*:de,ab,ti OR crossover*:de,ab,ti OR ((cross NEXT/1 over*):de,ab,ti) OR placebo*:de,ab,ti OR ((doubl* NEAR/1 blind*):de,ab,ti) OR ((singl* NEAR/1 blind*):de,ab,ti) OR assign*:de,ab,ti OR allocat*:de,ab,ti OR volunteer*:de,ab,ti	2527911
7.	#5 AND #6	32676
8.	#4 AND #7	743
9.	'phase 3 clinical trial (topic)'/exp	36043
10.	#6 AND #9	22280
11.	#4 AND #10	354
12.	#11 AND #12	5
13.	#8 OR #13	748

© 2021 Mohyuddin GR et al. *JAMA Network Open.*
